# Perceived risk for falls and decision-making in riding raised ramps in mountain biking: a pilot study

**DOI:** 10.3389/fpsyg.2023.1243536

**Published:** 2023-12-12

**Authors:** Emily P. Chilton, Stephen N. Robinovitch

**Affiliations:** Injury Prevention and Mobility Laboratory, Department of Biomedical Physiology and Kinesiology, Simon Fraser University, Burnaby, BC, Canada

**Keywords:** bicycling, risk-taking, risk perception, falls, injury, fear of falling, self-efficacy

## Abstract

Mountain biking (MTB) is a challenging activity where riders face constant decisions on whether to attempt technical paths or features (e.g., wooden ramps and jumps) that pose risk for falls and injuries. Risk homeostasis theory posits that riders pursue an optimal non-zero level of risk that balances the rewards of attempting challenging features with the need to avoid unreasonable risk for injury. Little is known on how riders judge risk, and the level of risk that riders deem unacceptable. We conducted experiments with experienced MTB riders (*n* = 17) to examine how their willingness to ride raised wooden ramps depended on their perceived probability for falling (*P_f_*) and their perceived probability for injury in the event of a fall (*P_i_*) while riding the ramp. In one experiment, participants viewed ramps of varying widths and heights and described their willingness to ride each ramp, along with *P_f_* and *P_i_*. We found that *P_f_* and *P_i_* were independent predictors of willingness to attempt ramps. Moreover, the product P_f_*P_i_ (the perceived risk for injury in attempting the ramp) was a stronger predictor than *P_f_* or *P_i_* alone. In a second experiment, participants viewed ramps of different widths, and reported the maximum (threshold) height where they would ride each ramp, along with *P_f_* and *P_i_*. We found that P_f_*P_i_ at the threshold height, averaging 13%, did not vary with ramp width. We conclude that decisions on riding ramps are based on the product P_f_*P_i_. On average, riders refused to ride ramps when P_f_*P_i_ exceeded 13%.

## Introduction

Mountain biking (MTB) is a popular recreational sport associated with a high risk for falls and fall-related injuries (Nelson and McKenzie, 2011; Becker et al., 2013; Palmer et al., 2021). The risks for falls and injuries in MTB depend on complex interactions between intrinsic factors (such as rider ability and decisions on use of protective gear) and situational and environmental factors (such as speed and physical features of the path including grade, width and roughness). However, the most common self-reported cause of fall-related injuries in MTB is errors in judgment (Gaulrapp et al., 2001; Becker et al., 2013). MTB requires constant decision-making on path selection (Hagen and Boyes, 2016) that affects the rider’s risk for imbalance, falls and injuries. This includes deciding on whether to attempt advanced features such as rock faces, gap jumps and raised wooden ramps, where the balance challenges may be high and the consequences of falls can be severe.

Decisions by MTB riders in attempting challenging features are governed by perceived risk–benefit ratios. Attempting challenging paths and features is a source of motivation and satisfaction for MTB riders (Taylor, 2010; Kerr and Houge Mackenzie, 2012). However, safe participation in MTB requires that riders avoid paths or features that are likely to lead to serious injury. Risk homeostasis theory (Wilde, 1998) posits that MTB riders select paths or features to maintain a preferred level of risk that is non-zero, thereby optimizing the benefit of the activity. Furthermore, risk is given by the likelihood of an adverse event, multiplied by the consequences of that event (Duijm, 2015). In the case of falls, risk depends on the probability of losing balance and falling, multiplied by the probability for serious injury in the event of a fall. Previous studies suggest that each of these parameters contribute toward defining action boundaries in the context of falls. Jiang and Mark (1994) found that the maximum gap distance that participants were willing to step across decreased with increasing gap depth. Similarly, Pijpers et al. (2006) observed a decrease in perceived and actual reaching distances in rock climbing with increased height above the ground. In both studies, perceived action boundaries narrowed when the consequences of a potential fall were more severe.

However, little is known on the level of risk that experienced MTB riders deem to be acceptable. Riders may pursue features where there is a relatively high risk for falling, if the risk for injury from a fall is low. Conversely, riders may avoid features where there is a relatively low risk for falling, if the risk for injury from a fall are high. In the current study, we conducted experiments to examine how the perceived risks for falls and injuries govern MTB riders in deciding to ride wooden ramps of varying width and height above the ground (“skinnys”), a common trail feature in MTB. By modifying the width of the ramp, we influenced the risk for falling off the edge; by modifying the height, we influenced the risk for injury in the event of a fall. We hypothesized that the willingness of riders to attempt a given combination of ramp height and width would depend on their perceived probability of falling (*P_f_*) and their perceived probability of injury in the event of a fall (*P_i_*). We also hypothesized that riders would attempt ramps when the product of these two probabilities (P_f_*P_f_), which reflects the perceived risk for injury in attempting the ramp, remained below a non-zero threshold.

## Methods

### Participants

MTB riders (*n* = 17; six females and 11 males) were recruited through notices on social media and snowball sampling. To be included in the study, riders had to be fluent in English, 19 years of age or older, and active in MTB (having participated in at least three off-road MTB rides in the past 2 months). Furthermore, we excluded riders with current injuries that precluded their participation in MTB, and those with moderate to severe visual impairment based on a LogMAR test score > 0.48 in both eyes (Bailey and Lovie, 1976; World Health Organization, 2019). The experimental protocol was approved by the Research Ethics Board of Simon Fraser University. Data collection took place on the Simon Fraser University Burnaby Campus between July and November, 2022. Participants arrived on the day of the experiment with their own mountain bike, helmet and protective gear. Participants completed a questionnaire describing their age, sex, and MTB experience. The mean age of participants was 47.1 years (SD 14.9; range 19–69). On average, participants had 21.0 (10.3) years of MTB experience and engaged in MTB 103.8 (70.4) days per year. Participants reported an average of 24 (59) falls in the last 12 months, and five out of 17 participants sought medical treatment for fall-related injuries.

### Ramp assessments

**Figure 1:**
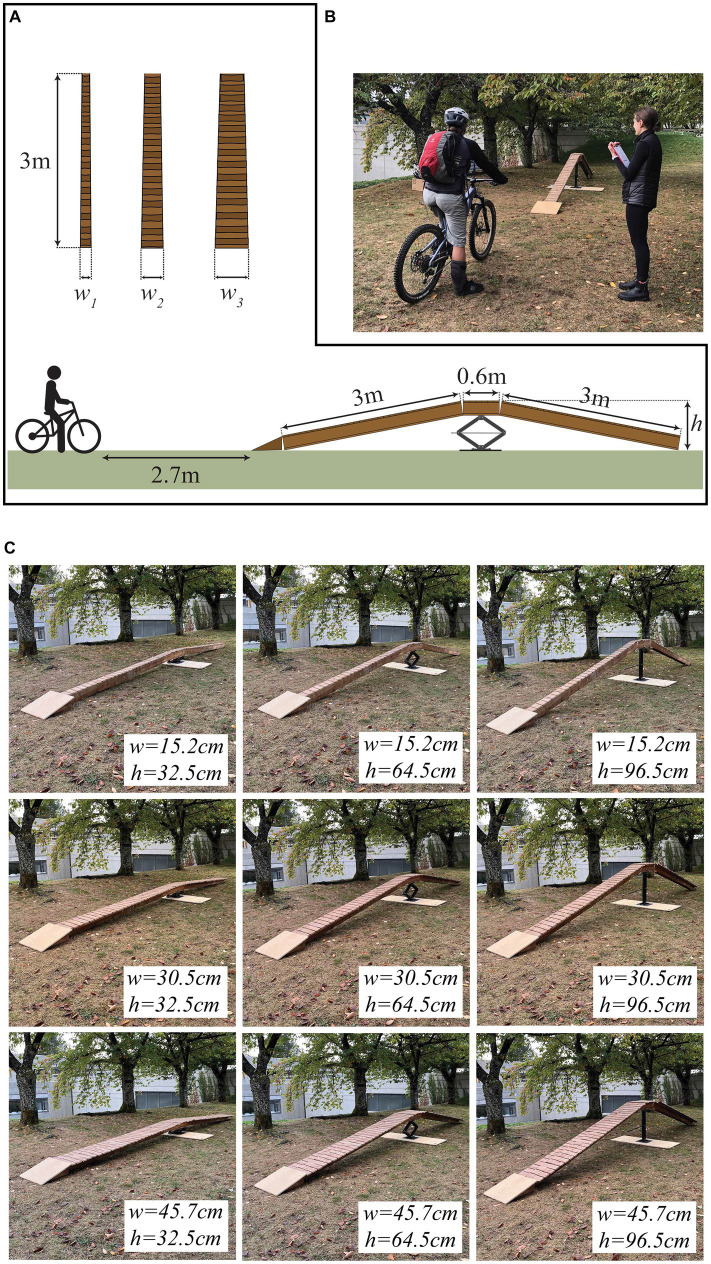
Experimental setup. **(A)** Ramp feature with adjustable width (*w*) and height (*h*). The ramp permitted continuous adjustments in height (between 32.5 and 96.5 cm) and discrete adjustments in width (between 15.2 and 61.0 cm, in increments of 7.6 cm). **(B)** Participants stood straddling their bicycle 2.7 m from the start of the ramp. **(C)** Nine ramp configurations were shown in discrete trials.

During the experiment, which was conducted on a level grass field, participants viewed and assessed their willingness to ride an A-frame wooden ramp for different values of ramp width and ramp height (Figure 1). The length of the ramp was 6.7 m. The height at the apex of the ramp could be adjusted continuously (via a scissor jack) between a minimum height of 32.5 cm, and a maximum height of 96.5 cm. The width of the ramp could be adjusted in increments of 7.6 cm between 15.2 and 45.7 cm. In all trials, participants stood straddling their bicycle 2.7 meters from the start of the ramp, and were instructed to imagine encountering the ramp while wearing their normal protective gear. Participants were not asked to ride the ramp, and were made aware of this fact before beginning their assessments.

Each participant completed “discrete trials” and “continuous trials.” In discrete trials, participants viewed a randomized sequence of nine ramp configurations (Figure 1C), that combined three different heights (32.5, 64.5, or 96.5 cm) and three different widths (15.2, 30.5, or 45.7 cm). For each configuration, the participant was instructed to describe their willingness to ride the ramp (yes or no), and to describe, on a scale of 0 to 100%, their perceived probability of falling off the ramp (*P_f_*), and their perceived probability of becoming injured in the event of a fall from the apex of the ramp (*P_i_*).

In continuous trials, we measured the maximum (threshold) ramp height, for a given ramp width, that the participant was willing to ride. Ramps of four widths (15.2, 22.9, 30.5, and 38 cm) were presented in a randomized order, and ramp height was slowly raised (from an initial value of 32.5 cm) via the scissor jack. Participants were instructed to say the word “stop” to indicate the maximum height they would be willing to attempt to ride on their bicycle (hereafter called threshold height), at which point we stopped raising the ramp. Once participants confirmed their estimate, they were instructed to imagine attempting to ride across the ramp, and to describe, on a scale of 0–100%, their perceived probability of falling off the ramp (*P_f_*), and their perceived probability of becoming injured in the event of a fall from the apex of the ramp (*P_i_*). In analysis of data from continuous trials, we excluded five participants whose threshold height was outside the attainable range of ramp height (i.e., below 32.5 cm or above 96.5 cm).

### Measures of riding ability

We measured the ability of participants to cycle along a straight line in a test similar to that used by Fonda et al. (2015). In this “straight line test,” participants rode on a level cement surface, where six pairs of rubber cones were placed at one-meter intervals. The gap width between each pair of cones was 15.2 cm, equal to the narrowest ramp width included in the ramp assessments. Participants were instructed to try their best to ride between each pair of cones, without having their wheels touch a cone. Participants started from a stationary position four meters behind the first set of cones, and completed five repeated trials. Each trial was captured on video and scored independently by two raters (a member of the research team and a trained volunteer). The outcome was the number of “clean” trials (ranging from 0 to 5), where both front and rear wheels passed through the gap between each of the six pairs of cones, without contacting a cone. Percent agreement between raters was 82.4%.

### Statistical analyses

We used logistic regression to examine how willingness to ride ramps in discrete trials depended on ramp height, ramp width, perceived probability of falls (*P_f_*), perceived probability of injury in the event of a fall (*P_i_*), and the product P_f_*P_i_. In each model, we included participant code as a covariate. We also used logistic regression to examine how willingness depended on “straight line test” scores. We compared the predictive accuracy of the models based on sensitivity, specificity, and Akaike Information Criterion (AIC) values. We also used repeated-measures ANOVA to examine how *P_f_*, *P_i_*, and P_f_*P_i_ in discrete trials depended on ramp width and ramp height, and how threshold height, *P_f_*, *P_i_*, and P_f_*P_i_ in continuous trials depended on ramp width. For all analysis, we divided values of *P_f_* and *P_i_* by 100, to convert from percentages to proportions (ranging between 0 and 1), and calculated P_f_*P_i_ based on the proportions. All statistical tests were performed using JMPv15 for Windows (SAS Institute Inc., Cary, United States). Statistical significance was set *α* = 0.05 *a priori*.

## Results

### Discrete trials: effect of ramp dimensions on willingness to ride

**Figure 2:**
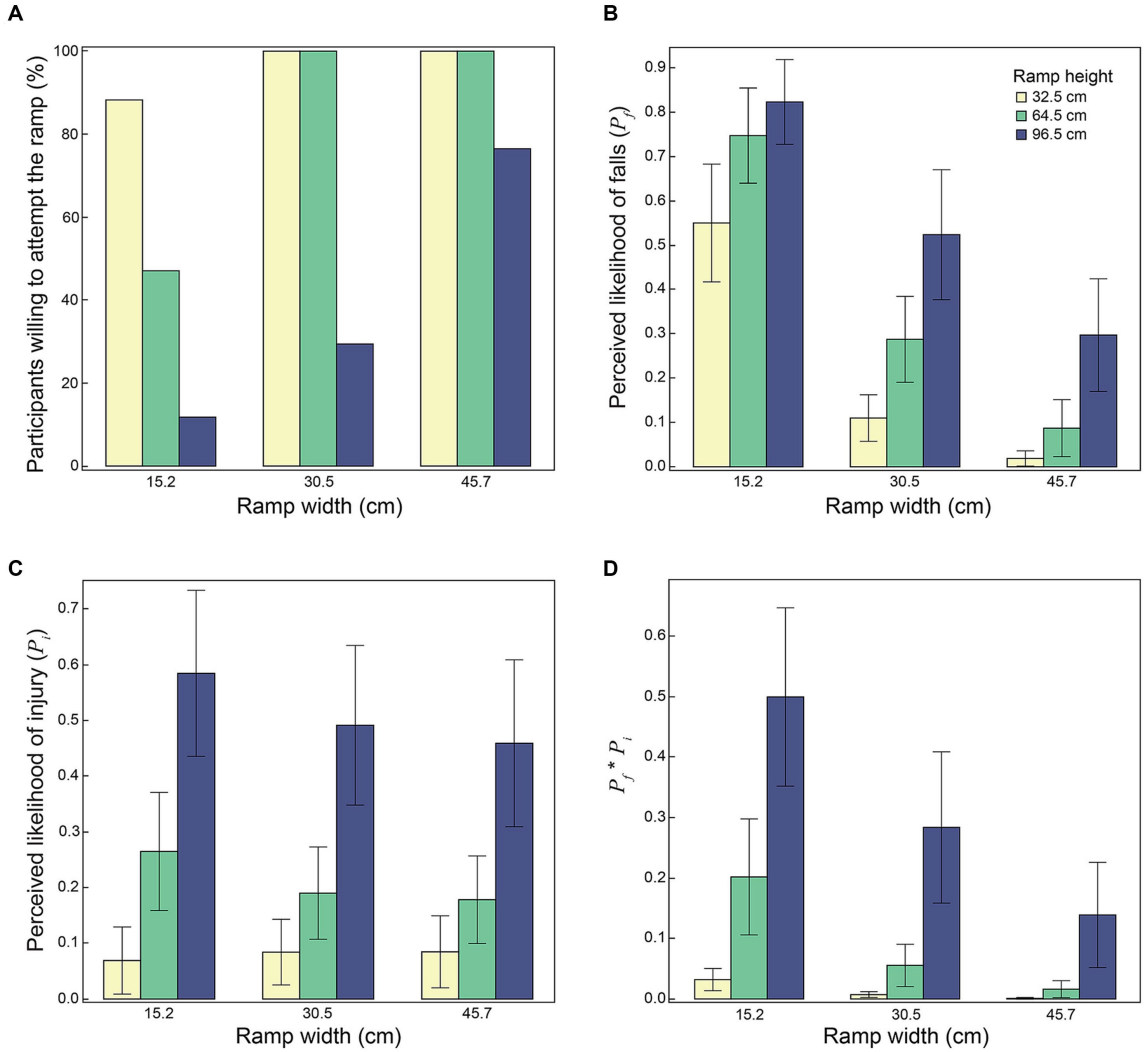
Results from discrete trials (*n* = 17), showing the effects of ramp width and ramp height on: **(A)** percent of participants willing to attempt the ramp; **(B)** mean values of perceived probability of falling off the ramp (*P_f_*); **(C)** perceived probability of injury in the event of a fall off the ramp (*P_i_*); and **(D)** the product P_f_*P_i_, which represents perceived probability of fall-related injury in attempting the ramp. **(B,C)** Show mean values and errors bars representing 95% confidence intervals.

In discrete trials, participants were willing to attempt an average of 6.5 out of the nine ramp configurations. While all participants were willing to attempt the widest (45.7 cm) ramp at the lowest (32.5 cm) height, only 11.8% of riders were willing to attempt the narrowest (15.2 cm) ramp at the highest (96.5 cm) height (Figure 2A).

Based on logistic regression (Table 1), willingness to attempt ramps increased with increasing ramp width (*p* < 0.0001) and decreased with increasing ramp height (*p* < 0.0001). A model that included both ramp height and ramp width provided 94.7% accuracy, 96.4% sensitivity and 90.5% specificity in predicting willingness to ride, and was more accurate than models that included only one of these variables (Supplementary Table S1). In this multivariable model, a 1 cm increase in ramp width was associated with a 46% increase in willingness to ride the ramp (OR = 1.46; 95%CI = 1.21–1.77). A 1 cm increase in ramp height was associated with a 21% reduction in willingness to ride the ramp (0.79; 0.71–0.89).

**Table 1:** Odds ratios on willingness to ride ramps in discrete trials (*n* = 17).

Predictor variables	OR (95% CI)	*p*-value
Model: ramp height, ramp width, participant		
Ramp width (1 cm increase)	1.46 (1.21–1.77)	<0.0001
Ramp height (1 cm increase)	0.79 (0.71–0.89)	<0.0001
Participant	…	<0.0001
Model: perceived probability for falling (*P_f_*), perceived probability for injury in falling (*P_i_*), participant		
*P_f_* (1% increase)	0.89 (0.84–0.94)	<0.0001
*P_i_* (1% increase)	0.88 (0.82–0.94)	<0.0001
Participant	…	0.043

### Discrete trials: effect of perceived risk for falls and injuries on willingness to ride

From logistic regression (Table 1), willingness to attempt ramps decreased with increases in *P_f_* (*p* < 0.0001) and with increases in *P_i_* (*p* < 0.0001). In a model that included *P_f_*, *P_i_*, and participant, an increase of 1% in *P_f_* associated with a 11% decrease in willingness to ride the ramp (0.89; 0.84–0.95), while an increase of 1% in *P_i_* associated with a 12% decrease in willingness to ride (0.88; 0.82–0.94). This multivariable model provided 92.1% accuracy, 95.5% sensitivity and 83.3% specificity in determining willingness to ride, and was more accurate than models that included only *P_f,_* or *P_i_* (Supplementary Table S1). Even greater accuracy (93.5%) was achieved by a model based on the product P_f_*P_f_ (Supplementary Table S1; Supplementary Figure S1).

### Discrete trials: effect of ramp dimensions on perceived risk for falls and injuries

Both ramp height and ramp width influenced *P_f_*, *P_i_* and the product P_f_*P_i_ (Figure 2). Each of *P_f_*, *P_i_* and P_f_*P_i_ associated negatively with ramp width (*p* < 0.001) and positively with ramp height (*p* < 0.001). Furthermore, there were significant interactions between ramp width and ramp height on *P_f_* (*p* = 0.004), *P_i_* (*p* = 0.006), and P_f_*P_i_ (*p* < 0.001). For the widest and lowest ramp, *P_f_* averaged 0.02, *P_i_* averaged 0.07, and P_f_*P_i_ averaged 0.0014. For the narrowest and highest ramp, *P_f_* averaged 0.82, *P_i_* averaged 0.58, and P_f_*P_i_ averaged 0.48.

### Continuous trials

**Figure 3:**
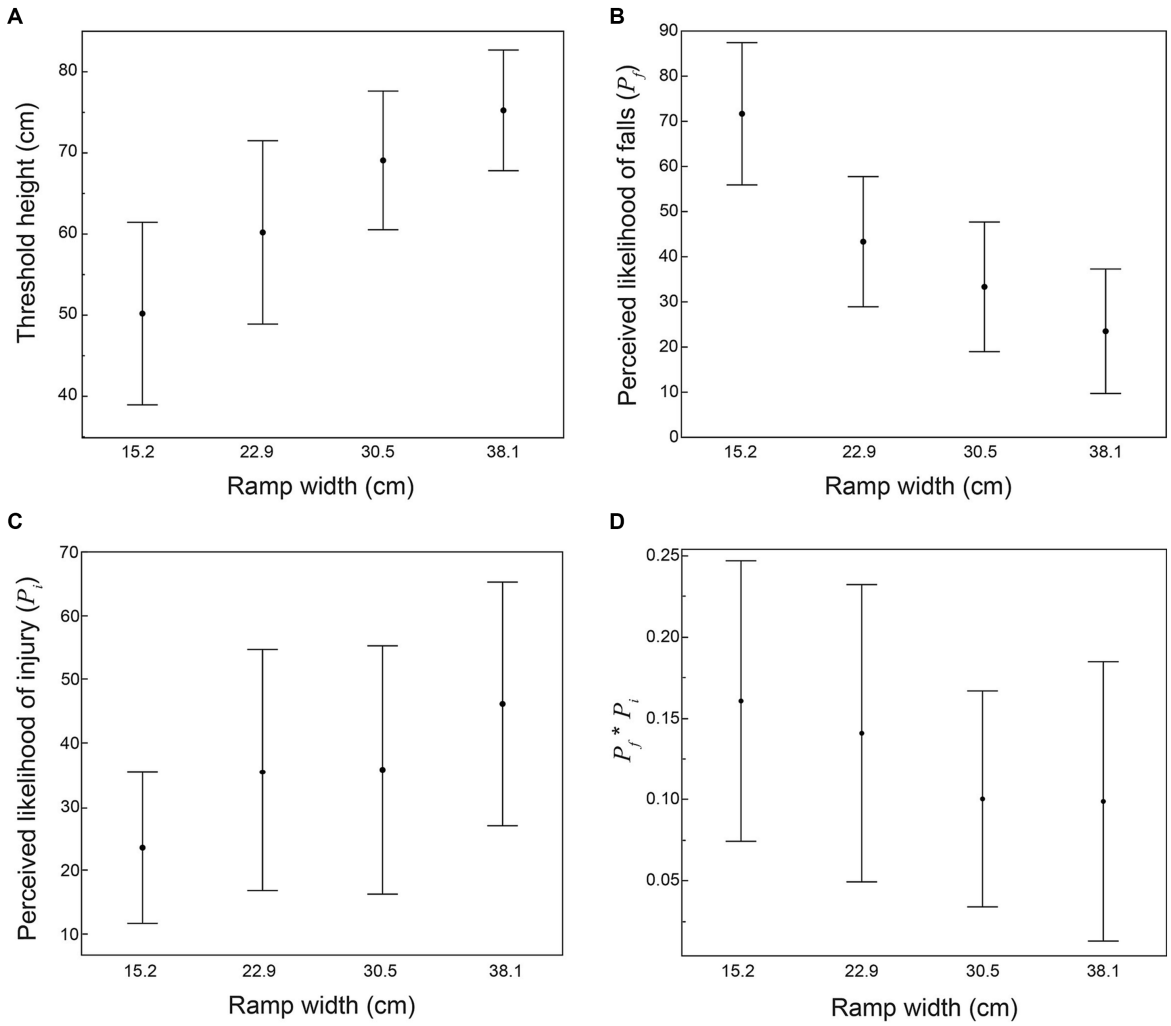
Results from continuous trials (*n* = 12), showing the effect of ramp width on: **(A)** threshold height; **(B)** perceived probability of falls from threshold height (*P_f_*); **(C)** perceived probability of injury in the event of a fall from threshold height (*P_i_*); and **(D)** the product of P_f_*P_i_ at threshold height. Graphs show means and errors bars representing 95% confidence intervals.

In continuous trials, the maximum (threshold) height where riders were willing to attempt to ride the ramp increased with increasing ramp width (*p* < 0.001 based on repeated-measures ANOVA; Figure 3A), averaging 50.2 cm for a 15.2 cm wide ramp, and 75.3 cm for a 38.1 cm wide ramp. The perceived probability of falling (*P_f_*) at the threshold height decreased with increasing ramp width (*p* < 0.001; Figure 3B), averaging 0.72 for a 15.2 cm wide ramp, and 0.24 for a 38.1 cm wide ramp. The perceived probability of injury from falling (*P_i_*) at the threshold height increased with increasing ramp width (*p* = 0.009; Figure 3C). *P_i_* averaged 0.24 for a 15.2 cm wide ramp, and 0.46 for a 38.1 cm wide ramp. The product P_f_*P_i_ (at threshold height) did not associate with ramp width (*p* = 0.068), and averaged 0.13 (95%CI = 0.09–0.16) (Figure 3D). The same value of P_f_*P_i_ = 0.13 predicted willingness to ride in discrete trials with 89% sensitivity, 86% specificity, 94% precision, and 88% accuracy (Supplementary Figure S1).

### Measures of riding ability

In the “straight line” tests of riding ability, the number of “clean” trials out of five (where both wheels passed through the 15.2 cm gap without touching any cone) averaged 1.24 (SD 0.83), and had a median value of 1 (8 participants), a maximum value of 3 (1 participant), and a minimum value of 0 (3 participants). This indicates that, on average, participants were incapable of riding within the 15.2 cm path 75.2% of the time {[1 − (1.24/5)]*100}. Based on logistic regression, willingness to ride ramps in discrete trials associated with the number of clean trials (OR 1.79; 95%CI: 1.11–2.88, *p* = 0.013). Based on linear regression, the threshold height in continuous trials did not associate with the number of clean trials (*p* = 0.31).

## Discussion

In MTB, riders are constantly faced with decisions on path selection that govern their risk for falls and injuries. Our study shows that decisions on riding wooden ramps of different heights and widths are based on the independent and interacting effects of perceived risk for falling (*P_f_*) and perceived risk for injury in the event of a fall (*P_i_*). Willingness to ride ramps was predicted more accurately by the combination of *P_f_* and *P_i_* than by *P_f_* or *P_i_* alone. Even greater accuracy was provided by a model based on the product P_f_*P_i_, which reflects the perceived probability of injury in attempting the ramp. In both discrete and continuous trials, riders were unwilling to ride ramps where P_f_*P_i_ exceeded about 0.13 (injury likely in one of eight attempts). This non-zero threshold value for P_f_*P_i_ is consistent with risk homeostasis theory (Wilde, 1998) which posits that riders pursue an optimal non-zero level of injury risk, to receive the benefits of attempting challenging paths and features (Taylor, 2010; Kerr and Houge Mackenzie, 2012).

We found that perceived risk for falls (*P_f_*) associated with both ramp width and ramp height. As expected, *P_f_* increased with decreases in ramp width. Less expected was the observation that *P_f_* also increased with increases in ramp height (for a given ramp width). We see three possible reasons why increases in ramp height led to loss of confidence in ability to ride the ramp without falling. First, the A-frame design of our ramp required participants to judge their ability to ride three sections of the ramp: ascending, level and descending. Increasing the ramp height from 32.5 to 96.5 cm caused the angle to increase from about 6–18 degrees. The change in angle may have influenced the perceived challenge of riding the ramp without falling. Second, the effect of ramp height on perceived risk for falling may reflect the influence of height-related “postural threat” on the nature and effectiveness of balancing strategies. Previous studies have shown that standing or walking on a raised platform (versus level ground) influences postural sway during quiet standing (Cleworth et al., 2016) and gait patterns during walking (Delbaere et al., 2009; Ma et al., 2021), presumably by increasing anxiety and reducing balance confidence. Third, participants may have perceived falls to be more likely at higher ramp heights due to the tendency for humans to overestimate the probability of catastrophic events and underestimate the probability of less serious events (Lichtenstein et al., 1978; Slovic et al., 1980).

We also found that perceived risk for injury in the event of a fall (*P_i_*) associated with both ramp width and ramp height. Not surprisingly, *P_i_* increased with increases in ramp height. More surprisingly, *P_i_* also increased with decreases in ramp width (for a given height). Narrowing of the ramp led to loss of confidence in ability to fall without injury. A possible explanation is that ramp width influenced potential landing strategies in the event of a fall (e.g., stepping onto the ramp with one or both feet), and thus the perceived risk for injury in the event of a fall.

Our study had important limitations. First, the adjustable ramp feature was restricted to a limited range of heights and widths. In continuous trials, five of the 17 participants were willing to ride at least one ramp width at the maximum height (96.5 cm). Future studies should employ higher and skinnier ramps to measure threshold heights for a wider range of riders. Second, we conducted all trials on a grassy field, which provided a relatively safe landing surface, when compared to the rocks, roots, and logs often encountered on MTB trails. Future studies should explore how the nature of the landing surface affects the outcomes from our study. Third, for safety reasons, participants did not ride the ramps, and were made aware of this fact while making their assessments. Not being required to ride the ramp may have caused participants to overstate their confidence in riding ramps. However, in the straight-line test of riding ability, participants were incapable of riding within the 15.2 cm path 75.2% of the time. This number aligns strongly with, and supports the accuracy of their claimed probability for falling, which averaged 71% (95%CI = 64–77) in discrete trials for the 15.2 cm ramp width. Furthermore, participant score on the straight-line test associated with willingness to attempt ramps in discrete trials. Therefore, we see little reason for questioning the validity and accuracy of the ramp assessments in our study.

Finally, the sample used in this exploratory study prevented meaningful examination of the effects on decision-making of variables such as age, sex, gender, and history of MTB-related falls and injuries. Previous studies have found that MTB riders who were younger and/or male were more likely to ride difficult trails (Fruhauf et al., 2020). However, female MTB riders experienced higher rates of serious injury than male riders (Kronisch and Pfeiffer, 2002; Carmont, 2008; Nelson and McKenzie, 2011). More research is needed to understand how demographic variables (e.g., age, sex, gender), psychological propensities (e.g., risk-taking and sensation seeking) and history of fall-related injuries affect decision-making in MTB.

In conclusion, MTB riders decided to attempt ramps of different widths and heights based on their perceived risk for falling and their perceived risk for injury in the event of a fall, such that the product of these two risks—the perceived probability for injury in attempting the ramp—remained below 0.13. By showing how MTB riders judge risks and select paths according to these risks, our results may inform improvements in trail design, trail classification, and rider education. Moreover, reasonable choices on path selection are essential to falls management and safe participation in a wide range of pursuits, from skiing, snowboarding, mountain climbing, hiking, and skateboarding, to rising from a chair or walking for the physically impaired. While our results are specific to riding wooden ramps in MTB, our methods provide an approach that can be translated for exploring whether similar threshold values of P_f_*P_i_ govern decision-making in other contexts.

## Data availability statement

The raw data supporting the conclusions of this article will be made available by the authors, without undue reservation.

## Ethics statement

The studies involving humans were approved by the Research Ethics Board of Simon Fraser University. The studies were conducted in accordance with the local legislation and institutional requirements. The participants provided their written informed consent to participate in this study. Written informed consent was obtained from the individual(s) for the publication of any potentially identifiable images or data included in this article.

## Author contributions

EC took a lead role in data collection and data analysis, generated a first draft of the manuscript, and participated in the experimental design and editing of the manuscript. SR participated in the experimental design, data collection, data analysis, preparation, and review of the manuscript. All authors contributed to the article and approved the submitted version.

## Supplementary material

Supplementary TABLE S1 Accuracy of logistic regression models in predicting willingness to ride ramps in discreet trials (*n* = 17).
