# Supplementary material for: Perceived risk for falls and decision-making in riding raised ramps in mountain biking: a pilot study
Source: Front Psychol. 2023 Dec 12;14:1243536. doi: 10.3389/fpsyg.2023.1243536 (PMC10773584; doi:10.3389/fpsyg.2023.1243536)
Supplement: Supplementary TABLE S1 — Accuracy of logistic regression models in predicting willingness to ride ramps in discreet trials (n = 17). [file Data_Sheet_1.PDF]

Supplementary Table 1. Accuracy of logistic regression models in predicting willingness to ride ramps in discreet trials (n=17).

**Model**	**Sensitivity (%)**	**Specificity (%)**	**Precision (%)**	**Accuracy (%)**	**AIC**
Ramp height, participant	87.4	73.8	87.4	83.7	141.2
Ramp width, participant	89.2	50.0	89.1	78.4	168.0
Ramp height, ramp width, participant	96.4	90.5	96.4	94.7	76.6
Perceived probability for falling (P_f), participant	92.7	81.0	92.8	89.5	120.6
Perceived probability for injury in falling (P_i), participant	90.1	78.6	91.7	86.9	131.7
$P_{f,}$, P_i, participant	95.5	83.3	95.5	92.1	86.5
P_f*P_i, participant	95.5	88.1	95.5	93.5	83.1

Note: AIC = Akaike Information Criterion.
