# Supplementary figures and images for: Perceived risk for falls and decision-making in riding raised ramps in mountain biking: a pilot study
Source: Front Psychol. 2023 Dec 12;14:1243536. doi: 10.3389/fpsyg.2023.1243536 (PMC10773584; doi:10.3389/fpsyg.2023.1243536)

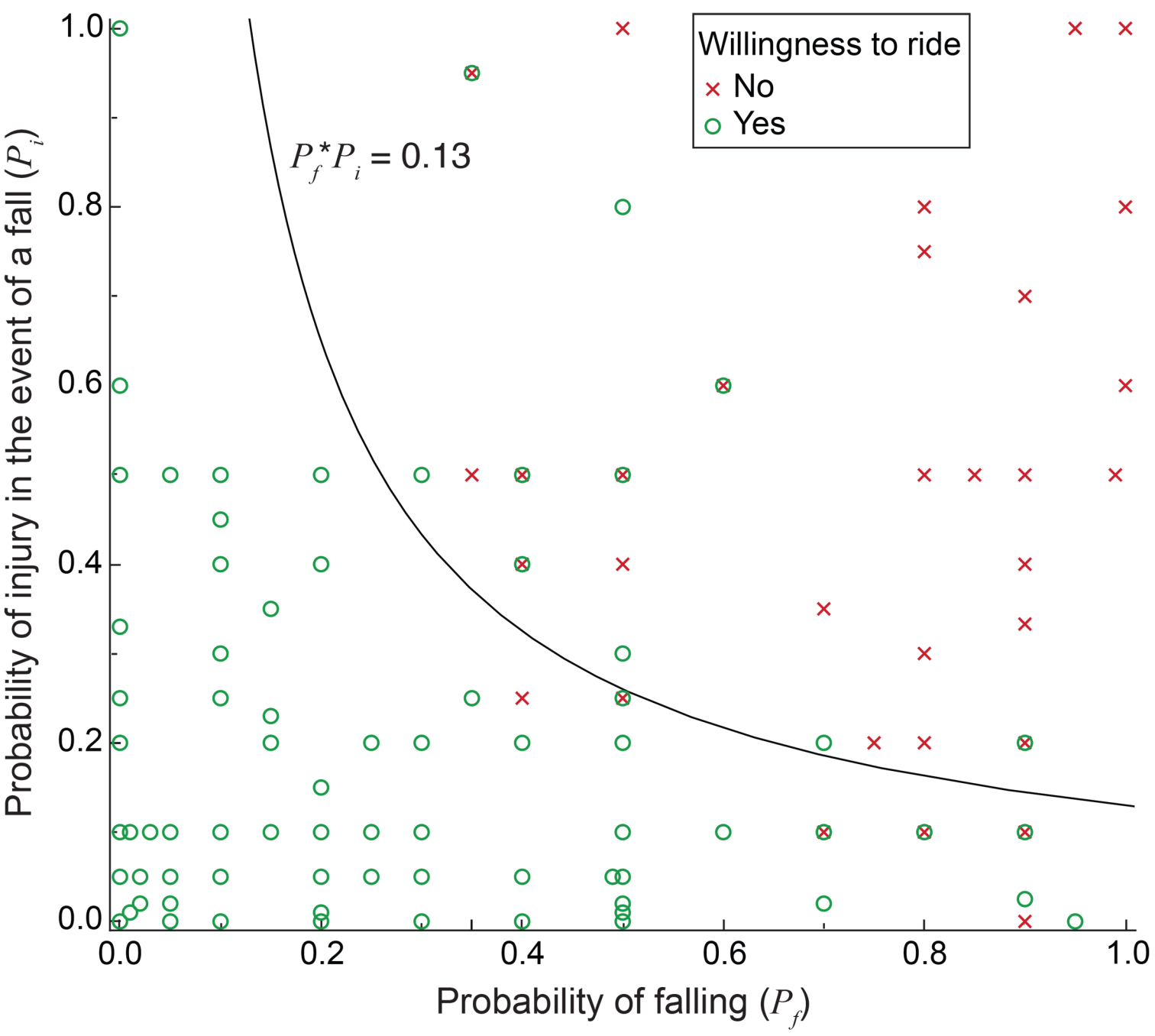

Supplement: Supplementary file 2 [file Data_Sheet_2.PDF]
